# Supplementary material for: Sensitivity of Rapid Antigen Testing and RT-PCR Performed on Nasopharyngeal Swabs versus Saliva Samples in COVID-19 Hospitalized Patients: Results of a Prospective Comparative Trial (RESTART)
Source: Microorganisms. 2021 Sep 9;9(9):1910. doi: 10.3390/microorganisms9091910 (PMC8464722; doi:10.3390/microorganisms9091910)
Supplement: Supplementary file 1 [file microorganisms-09-01910-s001.zip › microorganisms-1360212-supplementary/supplementary material/Figure S1.pdf]

CHUV
centre hospitalier universitaire vaudois
Institut de Microbiologie

Procedure for collecting saliva for COVID-19 saliva test (RESTART Study)

Before the test:

Make sure the patient will not eat, drink, brush his/her teeth or smoke at least 20 minutes (ideally 1 hour) before taking the sample.

How to perform the test:

1. When you are ready to perform the test take the cotton swab out of its pouch.
2. Rub with the end of the cotton swab and for at least 20 seconds the inside of patient's mouth by following the following sequence: internal surface of the cheeks on both sides → upper and then lower gingiva→over- and under- the tongue→ hard palate. Make sure you have put enough saliva on the cotton swab.

After the test:

1. Once the sample has been taken, place the swab in the tube, locating the breakable area (**figure S1A**).
2. Break the swab at the breakable part (**figure S1B**).
3. Close the cap properly and shake the tube 2-3 times to mix the sample well with the liquid in the tube (**figure S1C**).
4. Place the protected tube in the plastic sleeve (**figure S1D**), then close it and send it to Microbiology laboratory with patient's details and the service's label (**figure S1E**).

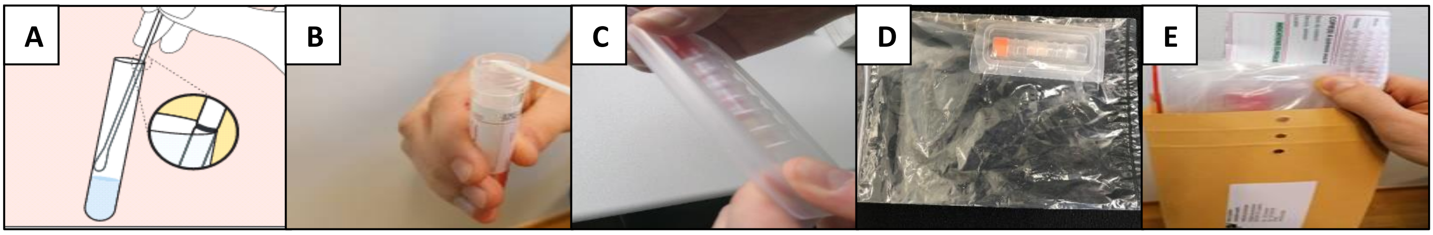

Figure S1: Sending sample to microbiology laboratory procedure.

If you have any questions, please contact the COVID microbiology line on 021/314 59 99 (8 am-10pm, 7 days a week)
