# Supplementary material for: Sensitivity of Rapid Antigen Testing and RT-PCR Performed on Nasopharyngeal Swabs versus Saliva Samples in COVID-19 Hospitalized Patients: Results of a Prospective Comparative Trial (RESTART)
Source: Microorganisms. 2021 Sep 9;9(9):1910. doi: 10.3390/microorganisms9091910 (PMC8464722; doi:10.3390/microorganisms9091910)
Supplement: Supplementary file 1 [file microorganisms-09-01910-s001.zip › microorganisms-1360212-supplementary/supplementary material/Table S1_In vitro evaluation of dilution effect.pdf]

Table S1: Limit of detection (LoD) for dry” versus “wet” swab procedure during the *in vitro* simulation experiment.

Type of swab	Type of test	Dil.1	Dil.2	Dil.3	Dil.4	Dil.5	Dil.6	Dil.7
“Dry” – Series 0	One Step Immunoassay Exdia®	P	P*	N	N	N	N	N
	Standard Q® Roche	P	P	P	N	N	N	N
	Cobas 6800 (Ct gene RDRP)	19.5	22.3	24.8	27.9	30.8	33.2	35.0
	Cobas 6800 (Ct gene E)	19.3	22.2	25.0	27.7	30.8	33.3	35.7
“Wet” – Series A	One Step Immunoassay Exdia®	P*	N	N	N	N	N	N
	Standard Q® Roche	P	P	N	N	N	N	N
	Cobas 6800 (Ct gene RDRP)	24.9	27.6	30.7	32.9	35.4	36.7	37.8
	Cobas 6800 (Ct gene E)	24.7	27.4	30.8	33.2	35.5	39.5	39.6
“Wet” – Series B	One Step Immunoassay Exdia®	P*	N	N	N	N	N	N
	Standard Q® Roche	P	N	N	N	N	N	N
	Cobas 6800 (Ct gene RDRP)	24.4	27.5	30.4	33.0	34.7	37.1	N
	Cobas 6800 (Ct gene E)	24.3	27.3	30.4	33.3	35.0	39.7	N

Series 0 was considered the internal reference for LoD, as if it corresponded to a dry swab. Series A and B were obtained after inoculating a swab, previously immerged in the corresponding tube from series 0, inside VTM tubes, thus simulating the process of wet swab. RT-PCR with Cobas 6800 was used as molecular confirmation and Ct calculation (Dil.= dilution; P= Positive test; N= Negative test; *= low positivity).
