# Supplementary figures and images for: Sensitivity of Rapid Antigen Testing and RT-PCR Performed on Nasopharyngeal Swabs versus Saliva Samples in COVID-19 Hospitalized Patients: Results of a Prospective Comparative Trial (RESTART)
Source: Microorganisms. 2021 Sep 9;9(9):1910. doi: 10.3390/microorganisms9091910 (PMC8464722; doi:10.3390/microorganisms9091910)

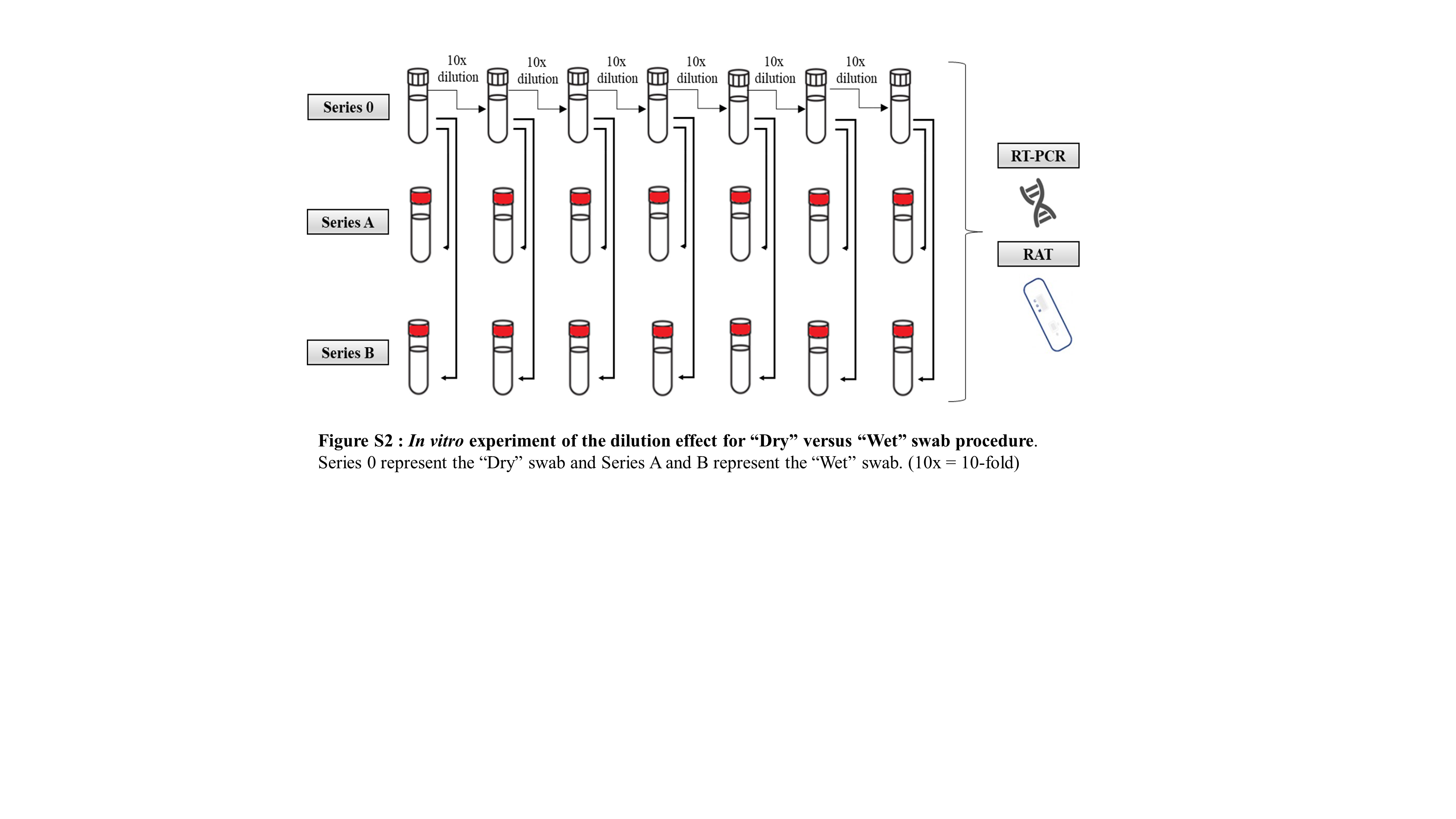

Supplement: Supplementary file 1 [file microorganisms-09-01910-s001.zip › microorganisms-1360212-supplementary/supplementary material/Figure S2.tif]

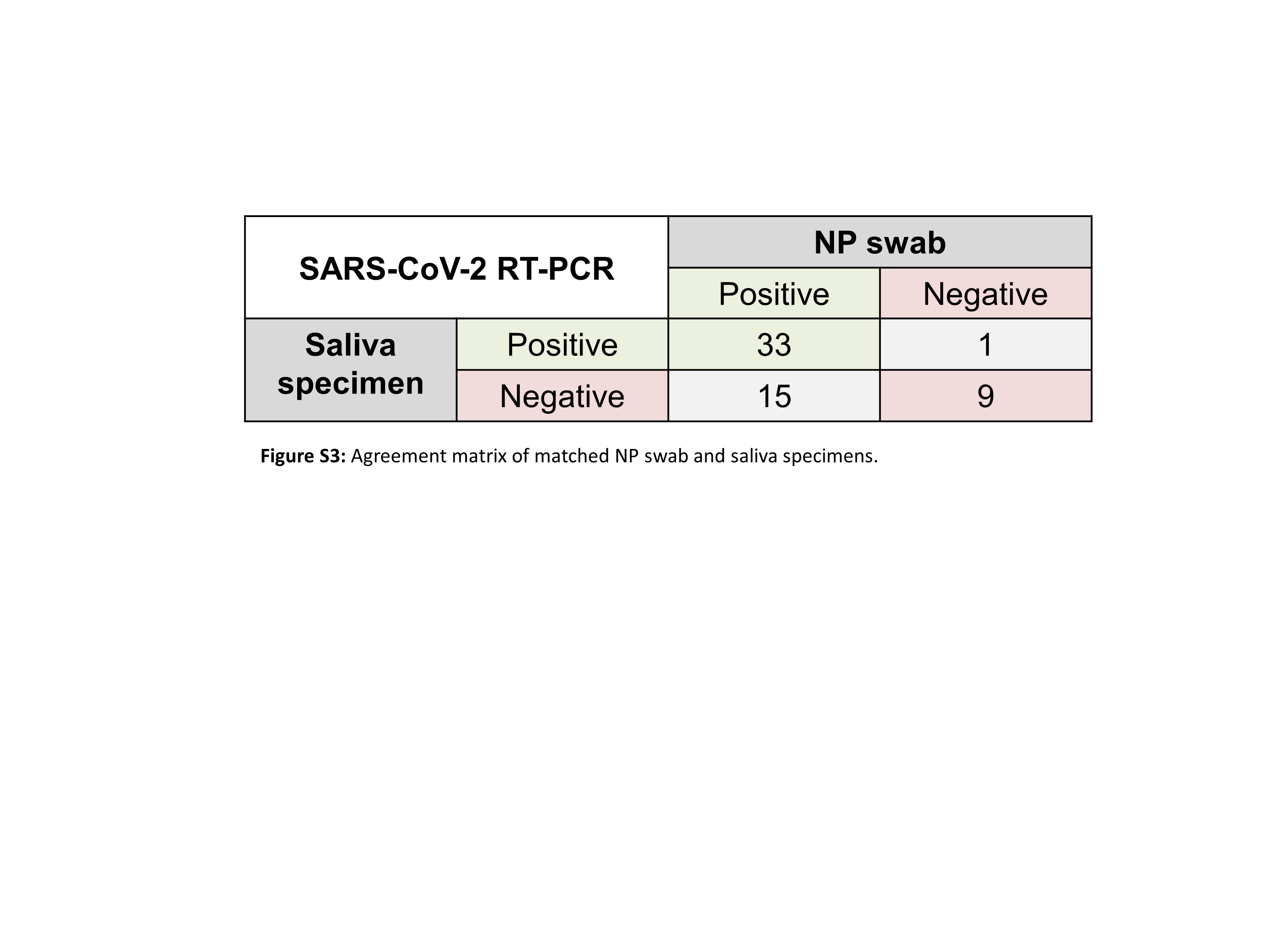

Supplement: Supplementary file 1 [file microorganisms-09-01910-s001.zip › microorganisms-1360212-supplementary/supplementary material/Figure S3.tif]

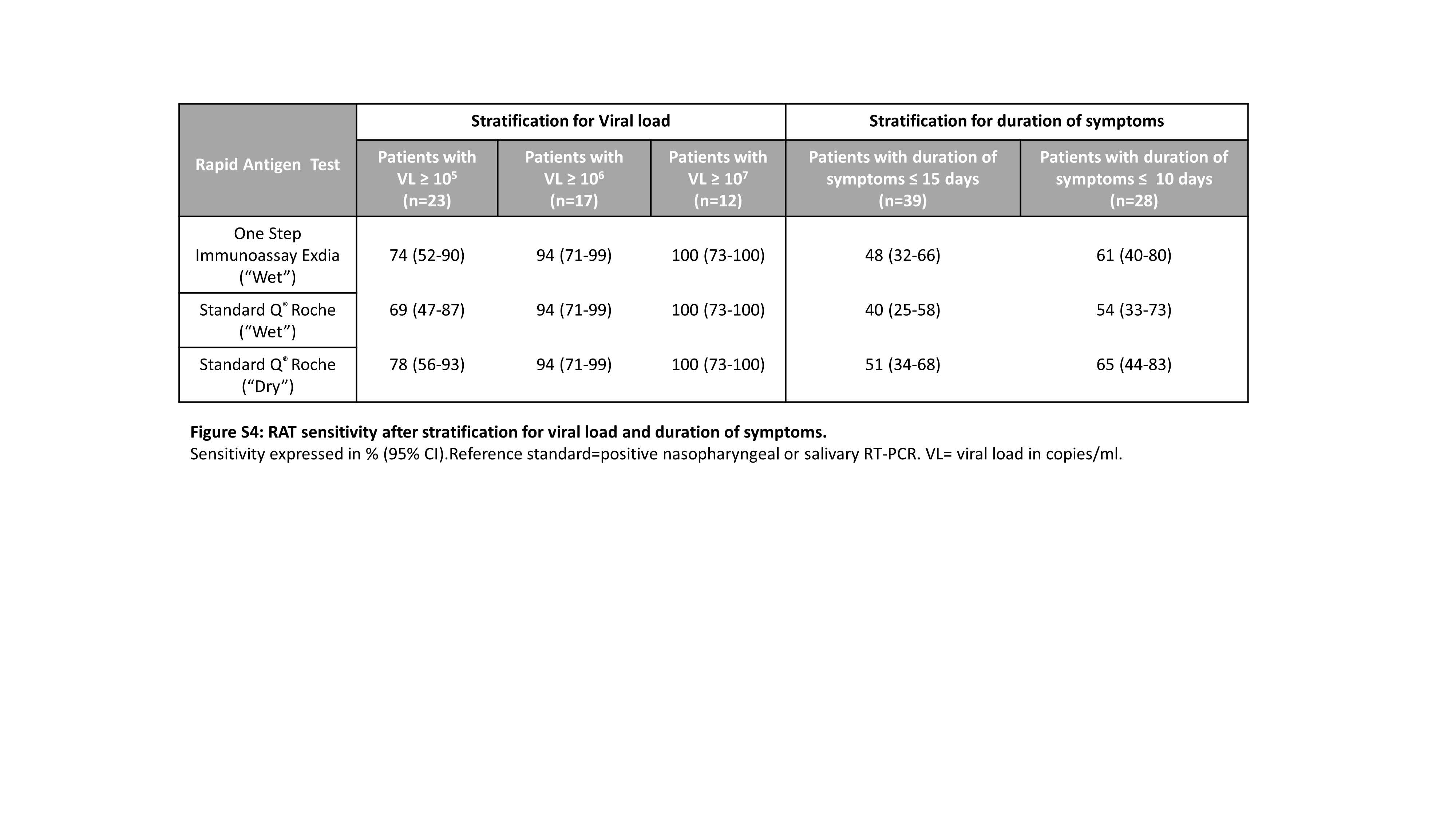

Supplement: Supplementary file 1 [file microorganisms-09-01910-s001.zip › microorganisms-1360212-supplementary/supplementary material/Figure S4.tif]
